# A dose-response characterization of transcranial magnetic stimulation intensity and evoked potential amplitude in the dorsolateral prefrontal cortex

**DOI:** 10.1038/s41598-023-45730-y

**Published:** 2023-10-30

**Authors:** Louisa Krile, Elnaz Ensafi, Jaeden Cole, Mah Noor, Andrea B. Protzner, Alexander McGirr

**Affiliations:** 1 Department of Psychology, University of Calgary, Calgary, AB Canada; 2 Department of Psychiatry, University of Calgary, 3280 Hospital Drive NW, TRW-4D68, Calgary, AB T2N 4Z6 Canada; 3 Hotchkiss Brain Institute, University of Calgary, Calgary, AB Canada; 4 Mathison Centre for Mental Health Research and Education, Calgary, AB Canada

**Keywords:** Neuroscience, Physiology

## Abstract

By combining transcranial magnetic stimulation (TMS) with electroencephalography, human cortical circuits can be directly interrogated. The resulting electrical trace contains TMS-evoked potential (TEP) components, and it is not known whether the amplitudes of these components are stimulus intensity dependent. We examined this in the left dorsolateral prefrontal cortex in nineteen healthy adult participants and extracted TEP amplitudes for the N40, P60, N120, and P200 components at 110%, 120%, and 130% of resting motor threshold (RMT). To probe plasticity of putative stimulus intensity dose-response relationships, this was repeated after participants received intermittent theta burst stimulation (iTBS; 600 pulses, 80% RMT). The amplitude of the N120 and P200 components exhibited a stimulus intensity dose-response relationship, however the N40 and P60 components did not. After iTBS, the N40 and P60 components continued to exhibit a lack of stimulus intensity dose-dependency, and the P200 dose-response was unchanged. In the N120 component, however, we saw evidence of change within the stimulus intensity dose-dependent relationship characterized by a decrease in absolute peak amplitudes at lower stimulus intensities. These data suggest that TEP components have heterogeneous dose-response relationships, with implications for standardizing and harmonizing methods across experiments. Moreover, the selective modification of the N120 dose-response relationship may provide a novel marker for iTBS plasticity in health and disease.

## Introduction

Concurrent transcranial magnetic stimulation and electroencephalography (TMS-EEG) is a powerful and non-invasive tool used to probe cerebral cortex physiology in health and disease. TMS-evoked potentials (TEPs), the time-locked signals elicited by single TMS pulses delivered to the cortex, are indicative of the underlying neuroanatomy and neurophysiology^1,2^. These negative and positive inflections of the electrical trace represent the summation of excitatory and inhibitory neurotransmission induced by stimulation^3–5^ and inform function and connectivity of the target site by characterizing the spatial and temporal spread of cortical activation^6,7^. In cortical regions that do not have a directly measurable functional output, such as the prefrontal cortex, TMS-EEG provides a unique lens into human cortical physiology.

Although TMS-EEG is a rapidly growing field with increasing technical rigor and practical application, there are several aspects of TMS-EEG that remain unstandardized. Dosing of the TMS pulse is one of the most crucial, yet there is substantial variability in the intensity of stimuli used in TMS-EEG experiments. Intensity of the TMS pulse is typically defined relative to a motor cortex output, such as the resting motor threshold (RMT). However, uniformity is lacking, as studies have used varying intensity ranging from 80 to 120% of motor threshold to explore cortical excitability^8–13^. Further, in TMS-EEG studies, RMT itself is variably defined as the intensity required to elicit a 0.05 mV or 1 mV motor-evoked potential from either the first dorsal interosseous or the abductor policis brevis^14–17^. It is possible that TEP findings vary between studies because of the stimulus intensity selected and, crucially, because TEP components may not all exhibit the same stimulus intensity dose-response features.

Here, we focus on the dorsolateral prefrontal cortex (DLPFC), a cortical region that plays a critical role in cognitive control and mood regulation^18,19^. Structural and functional abnormalities within this region are associated with numerous psychiatric conditions, and TMS-EEG studies of the DLPFC have uncovered neurophysiological differences associated with disease and response to interventions. Notably, amplitudes of N40 and N120 TEP components appear to be larger in individuals with major depressive disorder, a finding that has been validated in both youth and adults^20,21^. Features of DLPFC components have also been linked to aging, mild cognitive impairment, Alzheimer’s disease, and schizophrenia^14,17,22,23^. The potential utility of TEP components as biomarkers of disorder is further reinforced by their malleability with TMS protocols that drive synaptic plasticity, including those that are used therapeutically, such as repetitive TMS and theta burst stimulation^24–28^. Increasingly, we recognize that dosage considerations, such as the length of an iTBS train, are important and may have unique effects on TEPs^27^. In addition, interpreting differences across studies depends on an understanding of stimulus intensity dose-response relationships within the region of interest^29,30^.

In a group of healthy adults, we systematically examine stimulus intensity dose-response relationships in TEP component amplitudes in the DLPFC by varying stimulus intensity relative to motor threshold. We then test whether these stimulus intensity dose-response relationships change after participants undergo a clinically used TMS protocol. We hypothesized that a) TEP component amplitudes are differentially responsive to varying stimulus intensities, and b) these stimulus intensity dose-response relationships are significantly altered by intermittent TBS, a protocol that modulates cortical excitability.

## Method

This study was approved by the University of Calgary Conjoint Health Research Ethics Board (REB20-2034) and was performed in accordance with relevant guidelines and regulations. All participants provided written informed consent.

### Participants

We recruited and tested 21 healthy participants. Data from two participants were excluded from analyses, as one participant informed us of a neurodegenerative diagnosis shortly after participation and another participant’s EEG data had quality issues. The final sample therefore consisted of 19 individuals (30.89 ± 8.35 years, 63% female). Exclusion criteria included pregnancy, unstable medical illness, significant neurological disorder, history of stroke, intracranial implant, cardiac pacemaker, implanted medication pump, substance use disorder, and the inability to refrain from alcohol consumption 24 h prior to participation.

### Procedure

**Figure 1:**
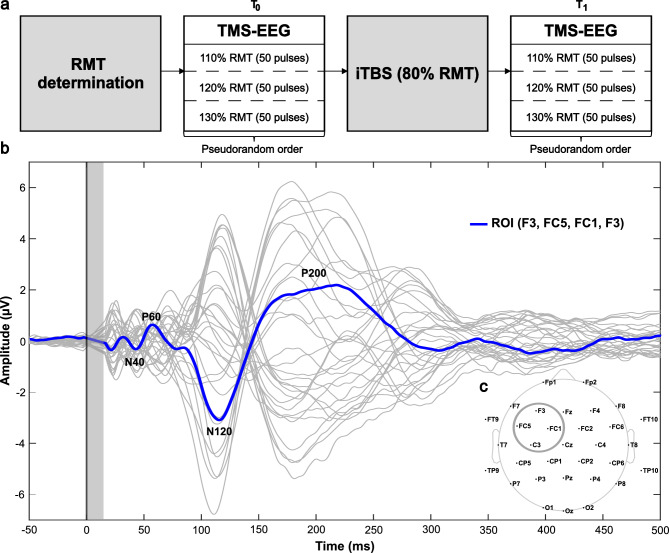
Diagram of the study procedure, region of interest electrodes, and illustrative TMS-evoked potential traces. (**a**) Schematic diagram of the study procedure. After determination of resting motor threshold (RMT), concurrent single-pulse transcranial magnetic stimulation and electroencephalography (TMS-EEG) was conducted at three different stimulus intensities (110%, 120%, and 130% RMT) before and after intermittent theta burst stimulation (iTBS) over the left dorsolateral prefrontal cortex. (**b**) TMS-evoked potentials from the average of the 120% RMT condition at baseline with components labelled. The vertical line at time = 0 marks the onset of the TMS pulse. The shaded grey bar represents the interpolated region (− 2 to 15 ms). The traces of each individual electrode are shown in light grey. The region of interest trace is superimposed in blue. c) Scalp topographic map with electrodes labelled. The average signal from the four circled electrodes (F3, FC5, FC1, C3) constitute the region of interest. Image generated using EEGLAB v14.1.1 (https://sccn.ucsd.edu/eeglab/download.php).

EEG data were collected concurrently with TMS delivery at three different stimulus intensities, 110%, 120%, and 130% RMT, before and after a course of iTBS (described below in Section “TMS”). These stimulus intensities reflect suprathreshold increments (relative to motor threshold) abutting the upper bound of stimulator output for most participants with our TMS-EEG configuration. This is consistent with TMS-electromyography (TMS-EMG) techniques where dose-response characterizations are more common. During TMS-EEG recordings, participants kept their eyes focused on a fixation point. To reduce auditory neural responses to the TMS coil discharge, participants wore ear plugs and noise-cancelling headphones playing a white noise recording. A memory foam pad compressible to 2 mm was placed on the coil to maximize comfort and minimize bone conduction. Each testing session lasted approximately 2 h. See Fig. 1a for a schematic diagram of the study procedure.

#### TMS

TMS was delivered using biphasic pulses with a MagPro X100 stimulator (MagVenture) and a Cool-B70 figure-eight coil held tangentially to the head and positioned 45 degrees laterally from the midline. RMT was determined with motor evoked potentials (MEPs) using an electromyographic electrode placed over the right first dorsal interosseous (FDI) muscle and a reference electrode placed over the first metacarpophalangeal joint. After locating the left motor cortex first dorsal interosseous muscle hotspot, RMT was defined as the minimum stimulus intensity required to generate MEPs at ≥ 0.05 mV on at least five of ten stimulations.

TMS was delivered to the left DLPFC defined using the Beam F3 method^31^ and the same location was used for both single-pulse TMS and iTBS. To ensure fidelity and consistency of coil placement across the experiment, Neuronavigation was utilized with a template brain registered to the participant’s anatomy (Visor2, ANT Neuro).

For single-pulse TMS, 150 pulses were delivered in pseudorandom order at 110%, 120%, and 130% RMT (50 pulses at each intensity, 0.2 Hz). iTBS (600 pulses in 20 trains of triplets at 50 Hz repeated at 5 Hz) was delivered at 80% RMT. Following iTBS, we repeated single-pulse TMS as a train of 150 pulses (0.2 Hz) where the intensity once again varied pseudorandomly between 110%, 120% and 130% RMT.

#### EEG

Continuous EEG data were collected using a BrainVision actiCHamp Plus system (Brain Products GmbH; Gilching, Germany) from an array of 32 TMS-compatible active slim electrodes (10–20 positioning; Cz as reference) at a rate of 10 kHz. DC-coupling of the amplifier was used to avoid amplifier saturation form the TMS artifact. Impedances of ≤ 10 kΩ were established prior to testing.

### TMS-EEG preprocessing

Data were preprocessed and analyzed offline using EEGLAB v14.1.1^32^ with the TESA v1.1.1^33^ plugin in MATLAB 2019a (MathWorks, Inc.). Bad channels were manually identified and spherically interpolated. Data were epoched − 1000 to 1000 ms around the TMS pulse and epochs were demeaned to remove DC offsets. Data between − 2 and 10 ms around the pulse were removed and cubically interpolated. These missing data points were interpolated prior to downsampling and filtering to avoid sharp transitions which can introduce ringing artifacts^33^. Data were then downsampled to 1000 Hz. Trials containing excessive noise were automatically detected, visually inspected, and confirmed artifacts were removed. Data values between − 2 and 10 ms were then replaced with constant amplitude data to improve ICA performance^33^. Pre- and post-iTBS data were concatenated and submitted to the FastICA algorithm using default settings. Individual components reflecting TMS decay artifact were detected using TESA *compselect*, visually inspected, and removed. The data removal window was then extended to − 2 and 15 ms around the pulse in order to remove residual TMS-evoked artifact^33^. Next, data were bandpass (fourth order, Butterworth, 1–80 Hz) and band-stop (second order, Butterworth, 58–62 Hz) filtered. A second round of FastICA was used to identify artifact associated with eye blinks, eye movements, muscle activity, electrode noise, and any remaining TMS decay artifact, and constant amplitude data were then linearly interpolated. Finally, data from the full epochs were re-referenced to the common average, baseline corrected (− 100 to 0 ms), and deconcatenated into separate conditions. See Supplementary Table S1 for information on trials, channels, and components removed for each condition.

#### TMS-evoked potential amplitudes

To assess the local response to TMS over the left DLPFC, our region of interest (ROI) was the average of four electrodes around the stimulation site (F3, FC5, FC1, C3). We focused on four components within the following time windows: the N40 (20–55 ms), P60 (40–90 ms), N120 (80–150 ms), and P200 (150–250 ms). Trials were averaged within each condition and TMS-evoked potential (TEP) amplitudes were calculated by averaging data ± 5 ms around the identified peak. As broad windows were used for finding peaks, each individual trace was manually checked before acceptance of peak latency and amplitude values. See Fig. 1b & c for a representative TEP trace and the topographic location of the electrodes included in the ROI, respectively.

### Statistics

Statistical analyses were carried out in IBM SPSS Statistics Version 25. To evaluate the dose-response function at the three stimulus intensities, separate general linear models (GLM) were conducted for each component using TEP amplitudes before iTBS with stimulus intensity (110%, 120%, 130%) as the within-subjects factor. General linear mixed effects models (GLMEM) with within-subjects intercepts were conducted for each component to evaluate changes in the dose-response function following iTBS, with intensity (110%, 120%, 130%) and timepoint (pre-iTBS, post-iTBS) as within-subjects factors. Post-hoc tests included pairwise parameter estimation for significant main effects and paired t-tests for simple main effects in significant interactions. Bonferroni corrections were applied to correct for multiple comparisons.

## Results

Demographic data are presented in Table 1. All four components of interest (N40, P60, N120, P200) were reliability elicited in all conditions, with latency values presented in Table 2, individual datapoints are presented in Supplementary Figs. S1 & S2, and topographic maps for grand average components are presented in Supplementary Fig. S3. TEP component peaks were not windowed based on the grand average because the latencies for these peaks were variable across individuals but highly consistent within individuals (Intra-class correlations: N40 = 0.84, P60 = 0.91, N120 = 0.92, and P200 = 0.84), consistent with considerable variability in the structure and function of the prefrontal cortex. Although the P30 component is commonly observed in TEP investigations of cortical excitability^34^, the magnitude of the TMS artifact at higher intensities impacted the reliability of this component and we excluded the P30 from analyses as it was not present in all conditions. As peak identification parameters (described in Section “TMS-evoked potential amplitudes”) did not always identify local peaks at every tested electrode in every participant, missing amplitudes were replaced using values from 15 unique datasets generated via multiple imputation (maximum 200 iterations, fully conditional specification, linear regression). In total, 3.95% of component amplitudes were not identified within the pre-defined windows, and Little’s MCAR test suggested that these values were missing at random (*p* > 0.999). For datasets with missing values, the median p-value generated by the imputation procedure is reported. See Supplementary Table S2 for outcomes of analyses with original and imputed datasets. Statistical significance of the effects described below were equivalent with the original and imputed datasets.

**Table 1:** Self-reported demographic characteristics of participants (*N* = 19).

Characteristic	n	%	Mean (SD)
Gender			
Female	12	63	–
Male	7	37	–
Age, years	–	–	30.89 (8.35)
Education, years	–	–	17.89 (3.26)
Ethnicity			
Asian	9	47	–
White	10	53	–

**Table 2:** Latency of TMS-evoked potential component amplitudes in each condition.

Component	Pre-iTBS			Post-iTBS		
	110%	120%	130%	110%	120%	130%
N40	41.13 (7.70)	41.35 (5.87)	41.06 (7.02)	35.89 (8.61)	41.12 (8.34)	41.33 (6.72)
P60	61.11 (13.55)	59.95 (12.16)	60.00 (9.84)	59.74 (10.12)	60.44 (10.88)	62.71 (11.74)
N120	113.47 (13.21)	113.00 (11.04)	114.47 (10.10)	112.63 (12.10)	114.84 (6.36)	111.11 (13.00)
P200	189.58 (25.23)	198.68 (28.72)	189.26 (25.56)	198.53 (27.15)	202.63 (27.08)	195.53 (29.97)

Values are presented as the mean (standard deviation) in milliseconds.

### TMS-evoked potential stimulus intensity dose-response

**Figure 2:**
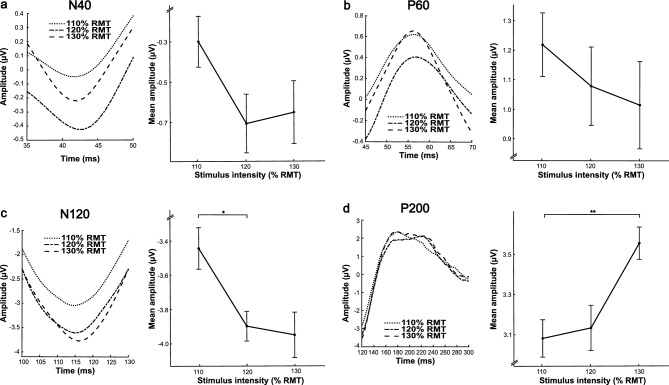
Grand average TMS-evoked potential traces and dose-response functions before intermittent theta burst stimulation. Grand average (*N* = 19 participants) TMS-evoked potential traces at the region of interest (F3, FC5, FC1, C3) and associated dose-response functions for the (**a**) N40, (**b**) P60, (**c**) N120^†^, and (**d**) P200 TMS-evoked potential components before intermittent theta burst stimulation over the left dorsolateral prefrontal cortex. It is important to note that grand average traces do not directly inform mean amplitudes as these are extracted on a per participant basis with variability in peak latency. Error bars represent ± 1 standard error of the mean. P-values correspond to general linear models. *p < .05. **p < .01. † One outlier was detected in the 130% condition but data quality did not justify exclusion from analyses.

Grand average TEP traces of each component for the three stimulus intensities pre-iTBS are presented in Fig. 2. Contrary to our hypotheses, no significant effects were identified for the N40 (*F*(2,36) = 1.59, *p* = 0.219, *ηp*^*2*^ = 0.081) and P60 (*F*(2,36) = 0.43, *p* = 0.656, *ηp*^*2*^ = 0.023) components (Fig. 2a & b). General linear models revealed a significant main effect of stimulus intensity for the N120 component (*F*(2,36) = 3.90, *p* = 0.029, *ηp*^*2*^ = 0.178; Fig. 2c). Pairwise parameter estimation showed that the mean amplitude for the 120% condition was significantly greater (i.e., more negative) than the 110% condition (*p* = 0.038, 95% C.I. = [0.02, 0.89]). There were no significant differences between the 130% condition and the 110% condition (*p* = 0.144, 95% C.I. = [− 0.12, 1.14]) and between the 130% and 120% condition (*p* > 0.999, 95% C.I. = [− 0.45, 0.55]). A significant main effect of stimulus intensity was also identified for the P200 component (*F*(2,36) = 4.88, *p* = 0.013, *ηp*^*2*^ = 0.213; Fig. 2d). Pairwise parameter estimation showed that the mean amplitude for the 130% condition was significantly greater than the 110% condition (*p* = 0.006, 95% C.I. = [0.12, 0.78]). There were no significant differences between mean amplitudes in the 110% and 120% conditions (*p* > 0.999, 95% C.I. = [− 0.43, 0.52]) and the 120% and 130% conditions (*p* = 0.073, 95% C.I. = [− 0.03, 0.83]).

#### Change in TMS-evoked potential stimulus intensity dose-response following iTBS

**Figure 3:**
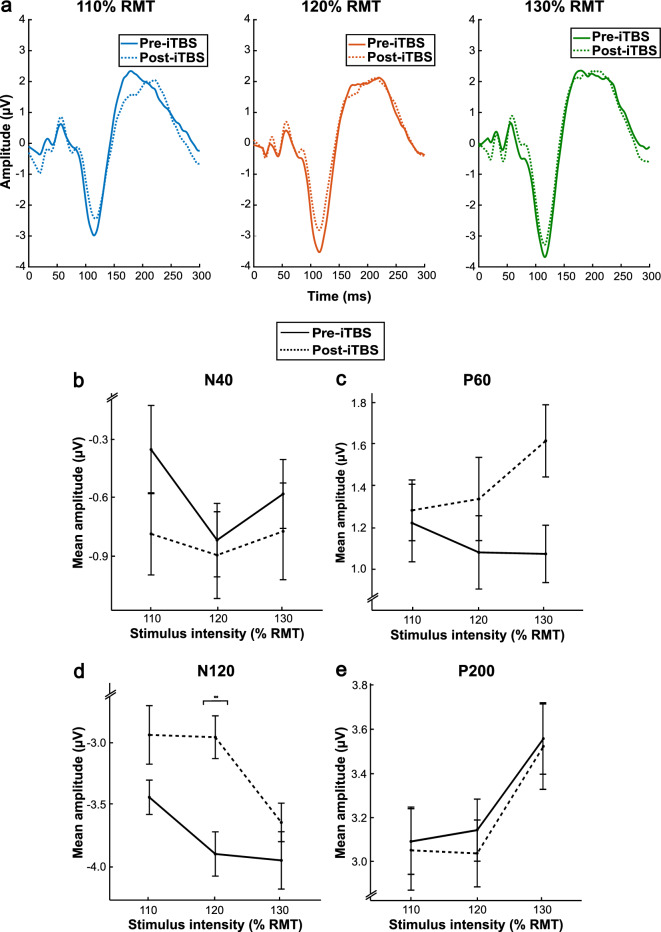
Grand average TMS-evoked potential traces and dose-response functions before and after intermittent theta burst stimulation. (**a**) Grand average (*N* = 19 participants) TMS-evoked potential traces at the region of interest (F3, FC5, FC1, C3) for each stimulus intensity (110%, 120%, 130% RMT) before and after intermittent theta burst stimulation over the left dorsolateral prefrontal cortex. (**b**–**e**) dose-response functions for the N40, P60, N120, and P200 TMS-evoked potential components, respectively, before and after intermittent theta burst stimulation over the left dorsolateral prefrontal cortex. It is important to note that grand average traces do not directly inform mean amplitudes as these are extracted on a per participant basis with variability in peak latency. Error bars represent ± 1 standard error of the mean. *P*-values correspond to general linear mixed effects models. *p < .05. **p < .01.

Grand average TEP traces at the three stimulus intensities pre- and post-iTBS are illustrated in Fig. 3a. Once again, there was no evidence of stimulus intensity dose-response relationships, and this was unchanged after iTBS for the N40 (*F*(2,36) = 0.62, *p* = 0.543, *ηp*^*2*^ = 0.033), P60 (*F*(2,36) = 0.86, *p* = 0.430, *ηp*^*2*^ = 0.046), and P200 (*F*(2,36) = 0.05, *p* = 0.956, *ηp*^*2*^ = 0.003) components (Fig. 3b,c & e). However, general linear mixed effects models revealed a significant intensity by time interaction for the N120 component (*F*(2,36) = 5.27, *p* = 0.010, *ηp*^*2*^ = 0.226; Fig. 3d). Paired t-tests showed that there was a significant decrease in the absolute mean amplitude for the 120% condition (i.e., more positive) from pre- to post-iTBS (*t*(18) = − 3.03, *p* = 0.007). There were no significant differences in mean amplitudes for the 110% (*t*(18) = − 1.62, *p* = 0.123) and 130% (*t*(18) = − 0.95, *p* = 0.354) conditions following iTBS.

## Discussion

In this study, we evaluated TEP component stimulus intensity dose-response relationships in the left DLPFC using three stimulus intensities and probed plasticity within these putative dose-response relationships. Contrary to our hypotheses, our analyses suggest that stimulus intensity dose-response relationships are not generalized in the DLPFC, and that increasing stimulus intensity selectively impacts later TEP components. Importantly, it is only a subset of TEP components, those with a pre-existing stimulus intensity dose-response relationship, that appear to be subsequently modifiable by iTBS.

We observed significant stimulus intensity dose-response relationships in the N120 and P200 components and, surprisingly, only the N120 dose-response relationship was modified by iTBS, suggesting it is sensitive to both the effects of varying intensity in single-pulse protocols and plasticity-inducing protocols. However, when the interest is on earlier TEP components, our data suggest that component amplitudes are consistent and reliable at lower stimulus intensities such that higher intensity TMS pulses, and their associated TMS-induced artifact, may not be necessary. Moreover, our data have important implications for the standardization of TMS-EEG stimulation parameters, particularly for the valid interpretation of the N120 and P200 peaks, and for the interpretation and synthesis of data with different methodologies.

TMS-evoked potentials in the DLPFC are canonically the N40, P60, N120 and the P200. Pharmacological studies have revealed different contributions to each of these, with the N40 representing an integration of GABA-A and NMDA receptor contributions^35,36^, the P60 reflecting glutamatergic AMPA receptor signalling^35^, the N120 reflecting GABA-A and GABA-B receptor signalling^35–39^ and the P200 reflecting the integration of multiple inputs as a global marker of excitability^40,41^. Accordingly, our data suggests that input–output functions in the DLPFC may be particularly relevant for the study of GABAergic signalling and plasticity.

The N120 component is also of clinical interest and has been implicated in neuropsychiatric conditions. For example, Noda and colleagues demonstrated that N120 amplitudes were significantly smaller in participants with schizophrenia compared with healthy controls^22^. Tallus and colleagues found that N120 amplitudes were significantly higher in symptomatic compared to asymptomatic mild traumatic brain injury patients and controls^42^. In a machine learning paradigm, Zhang and colleagues found that the N120 component was able to differentiate cognitively impaired participants from healthy controls with relatively high accuracy^17^. The N120 also plays a notable role in major depressive disorder, with Voineskos and colleagues demonstrating that amplitudes are significantly larger in adults with depression compared with healthy controls^21^, and Dhami and colleagues reproducing this effect in depressed youth^20^.

The N120 is also amenable to change with both single-session and long-term repetitive TMS protocols. For example, a 6-week randomized controlled trial of repetitive TMS over the left DLPFC in treatment-resistant depression resulted in decreased N120 amplitudes following active stimulation^28^. Chung and colleagues have demonstrated significant modulation of N120 amplitudes following iTBS over the left DLPFC in three different studies^24–26^. This effect of iTBS on N120 amplitudes has also been observed in primary motor cortex N120^43^. The implication of a stimulus intensity dose-response relationship for this GABAergic marker is very relevant, as GABAergic plasticity is hypothesized to underlie large scale network changes with rTMS^44^. Further, the iTBS protocol is thought to induce NMDA receptor dependent plasticity in part through GABA autoreceptor activation at the theta frequency^45,46^. Our data suggest that future studies examining the N120 component may benefit from incorporating stimulus intensity dose-response characterizations to maximize the interpretability of findings and the prognostic utility in therapeutic contexts. Further, we believe it is important to highlight the strength of the dose-response approach when characterizing cortical function, as it can reveal a shift in dose-response relationships whereas single intensities may not show the neuroplastic effects of plasticity protocols such as iTBS. We caution against interpreting that any one stimulus intensity is optimally suited to uncovering these effects based on statistical separation in post-hoc testing.

An important caveat to our work is the narrow range of stimulus intensities used, all of which were suprathreshold to an FDI motor threshold. It is possible that stimulus response relationships in the prefrontal cortex are observed at intensities that are inferior to motor threshold in earlier TEP components, in which case our data would instead reflect a ceiling effect once the neural activation threshold of 100 V/m is met^47^. This may be consistent with the sigmoidal function that emerges from TMS-EMG, where motor cortex neurons are recruited by an increasing stimulus intensity until a limit wherein expanding the volume of stimulated cortex no longer recruits additional neurons involved in the motor representation and an MEP plateau is reached^48^. While it is possible that DLPFC early components exhibit a dose-response that is saturated at suprathreshold intensities, in motor cortex subthreshold TMS pulses only elicit weak TEP waveforms^49^. We cannot exclude the possibility, however, that subthreshold pulses would reveal a stimulus-intensity dose-response in the DLPFC. There is considerable evidence that properties of sigmoidal TMS-EMG input–output curves are sensitive to iTBS effects^50–53^. Therefore, future research should evaluate the full range of TEP properties in DLPFC to determine whether a similar sigmoidal function is present and its practical applicability to plasticity-inducing protocols.

## Limitations

The auditory evoked potential typically manifests 100–200 ms post-stimulation, and despite the steps taken to mitigate this confound (i.e., ear plugs, noise-cancelling headphones playing white noise, and a foam pad placed between the coil and head), we cannot exclude the possibility that auditory effects contributed to our stimulus intensity dose-response findings^54–57^. It is also worth noting that we did not confirm effectiveness by asking participants if they could hear the coil discharge.

We were unable to reliably examine the P30 component, and the N40 and P60 peaks were small in amplitude and may have benefited from more trials to improve signal-to-noise ratio and peak estimation. However, the validity of a within-individual design including a pre-post iTBS comparison required balancing experimental length and the possibility of participant movement. Despite the use of neuronavigation to ensure accuracy and consistency of placement, subtle shifts would have compromised the interpretation of stimulation effects.

We determined stimulation intensity relative to first dorsal osseous RMT. However, we acknowledge that anatomical differences between prefrontal cortex and motor cortex (including but not limited to scalp-to-cortex difference and gyral configuration) introduce important structural and functional variability across participants. However, this is mitigated in part by the use of a within-subject design.

Lastly, informed by TMS-EEG dose-response relationship in motor cortex^49^, we did not include stimulus intensities subthreshold to FDI motor threshold, but it is possible that this precluded detecting stimulus intensity dose-response relationships in the N40 and P60 components.

## Conclusion

Using TMS-EEG to probe stimulus response relationships in the DLPFC, we show that TEP components do not all exhibit dose-response relationships to suprathreshold stimulation. Furthermore, our data suggest that neurostimulation, in this case iTBS, produces a selective shift in the stimulus intensity dose-response function only for the N120 component. Component amplitudes elicited at a single intensity should be interpreted with caution, and future studies of GABAergic signalling with TMS-EEG would benefit from incorporating stimulus intensity dose-response characterizations.

### Supplementary Information

Supplementary Information.

## Data Availability

Data will be made available from the corresponding author upon reasonable request.
