# Supplementary material for: A dose-response characterization of transcranial magnetic stimulation intensity and evoked potential amplitude in the dorsolateral prefrontal cortex
Source: Sci Rep. 2023 Oct 30;13:18650. doi: 10.1038/s41598-023-45730-y (PMC10616119; doi:10.1038/s41598-023-45730-y)
Supplement: Supplementary file 1 — Supplementary Information. [file 41598_2023_45730_MOESM1_ESM.docx]

**Supplementary Information**

**A dose-response characterization of transcranial magnetic stimulation intensity and evoked potential amplitude in the dorsolateral prefrontal cortex**

Krile, Louisa, BSc^1,3^

Ensafi, Elnaz, PhD^2,3,4^

Cole, Jaeden, BSc^2,3,4^

Noor, Mah, BSc^1^

Protzner, Andrea B., PhD^1,3,4^

McGirr, Alexander, MD PhD FRCPC^2,3,4^*

^1^ Department of Psychology, University of Calgary, Calgary, Alberta, Canada.

^2^ Department of Psychiatry, University of Calgary, Calgary, Alberta, Canada.

^3^ Hotchkiss Brain Institute, University of Calgary, Calgary, Alberta, Canada.

^4^ Mathison Centre for Mental Health Research and Education, Calgary, Alberta, Canada.

***Correspondence should be addressed to:**

Alexander McGirr

Department of Psychiatry

University of Calgary

3280 Hospital Drive NW, TRW-4D68

Calgary, AB

T2N 4Z6

Canada

[alexander.mcgirr@ucalgary.ca](mailto:alexander.mcgirr@ucalgary.ca)

**Supplementary Figure S1. Individual datapoints for each component and condition before iTBS.** Dose-response functions (black) and individual datapoints (grey) for the **a)** N40, **b)** P60, **c)** N120, and **d)** P200 TMS-evoked potential components before intermittent theta burst stimulation over the left dorsolateral prefrontal cortex.

**Supplementary Figure S2. Individual datapoints for each component and condition after iTBS.** Dose-response functions (black) and individual datapoints (grey) for the **a)** N40, **b)** P60, **c)** N120, and **d)** P200 TMS-evoked potential components after intermittent theta burst stimulation over the left dorsolateral prefrontal cortex.

**Supplementary Figure S3. Topographical surface voltage maps for each component and condition before and after iTBS.** Grand average voltage values were calculated using the following time windows for each component: N40 (30-50 ms), P60 (50-70 ms), N120 (100-130 ms), and P200 (160-240 ms). Images generated using EEGLAB v14.1.1 (https://sccn.ucsd.edu/eeglab/download.php).

| **Component** | **Pre-iTBS** | |  | | **Post-iTBS** | |
| --- | --- | --- | --- | --- | --- | --- |
| **Channels removed** |  | 0.65 [0-2] |  |  | 0.5 [0-2] |  |
| **Components removed (ICA1)** |  | 2.84 [1-7] |  |  | - |  |
| **Components removed (ICA2)** |  | 7.47 [3-11] |  |  | - |  |
|  | **110%** | **120%** | **130%** | **110%** | **120%** | **130%** |
| **Total trials** | 47.1 [40-49] | 47.5 [46-50] | 47 [44-50] | 47.1 [44-49] | 47.2 [44-50] | 47.1 [45-49] |

**Supplementary Table S1.** Channels removed, components removed, and trials remaining after preprocessing. Values are presented as the mean (range). - Indicates that conditions were concatenated prior to ICA.

| Imputation | Pre-iTBS Post-iTBS | | | |
| --- | --- | --- | --- | --- |
|  | **N40** | **P60** | **N40** | **P60** |
| Original | 1.45, .254 | .463, .633 | .184, .720^†^ | .251, .780 |
| 1 | 1.59, .219^*^ | .552, .581 | .406, .669 | 1.63, .211 |
| 2 | 2.01, .148 | .313, .733 | .357, .702 | 1.23, .304 |
| 3 | 1.23, .304 | .249, .781 | .824, .447 | .863, .430^*^ |
| 4 | 1.53, .231 | .170, .845 | .331, .720 | .730, .489 |
| 5 | 2.44, .101 | .695, .505 | 1.26, .295 | .714, .497 |
| 6 | .712, .498 | .427, .656^*^ | .312, .665^†^ | .497, .613 |
| 7 | 2.10, .137 | .589, .560 | 1.36, .269 | 1.71, .195 |
| 8 | 1.81, .179 | .828, .445 | .820, .449 | .898, .416 |
| 9 | 1.05, .362 | .690, .508 | .225, .800 | 1.19, .315 |
| 10 | 1.41, .258 | .244, .785 | .620, .543^*^ | .310, .674^†^ |
| 11 | 1.29, .289 | .236, .791 | 1.06, .358 | .641, .533 |
| 12 | 1.85, .172 | .287, .752 | .622, .543 | 1.01, .375 |
| 14 | 1.78, .183 | .668, .519 | 2.21, .138 | 1.32, .279 |
| 15 | 1.55, .227 | .256, .775 | .197, .822 | .669, .519 |

**Supplementary Table S2.** General linear model and general linear mixed effect model results for original and imputed datasets. Values are presented as the F-statistic, p-value. * Represents the median p-value of the imputed datasets. If the median p-value was present in more than one dataset, the smallest F-statistic was reported. † Indicates the sphericity assumption was violated (Mauchly’s Test of Sphericity *p* < .05) and Greenhouse-Geisser corrected p-values are reported.
